# Supplementary material for: Daily and Seasonal Variation in Light Exposure among the Old Order Amish
Source: Int J Environ Res Public Health. 2020 Jun 21;17(12):4460. doi: 10.3390/ijerph17124460 (PMC7344929; doi:10.3390/ijerph17124460)
Supplement: Supplementary file 1 [file ijerph-17-04460-s001.pdf]

Table S1. Relationship between spectral light exposure and photoperiod. Shows total (irradiance in Log µW/cm[2] summed over each min of exposure) and average irradiance (mean±SD) reported by Red, Green and Blue sensors for each analysis epoch tested under winter/spring (short) and summer (long) photoperiods. Conventions as in Table 1.

	Total Exposure (Log µW.cm²*min)				Average Exposure (Log µ.cm²)			
	Winter-Spring	Spring-Summer	Correlation with photoperiod		Winter-Spring	Summer	Correlation with photoperiod	
Epoch	Mean ± SD	Mean ± SD	r	*p*	Mean ± SD	Mean ± SD	r	*p*
24h Total (R)	4.69 ±0.28	5.22 ±0.26	**0.70**	**<0.001**				
24h Total (G)	5.01 ±0.30	5.53 ±0.27	**0.71**	**<0.001**				
24h Total (B)	4.63 ±0.27	5.10 ±0.27	**0.70**	**<0.001**				
Pre-Dawn (R)	1.94 ±0.70	1.05 ±1.32	**-0.58**	**<0.001**	0.03 ±0.5	-0.62 ±1.08	**-0.48**	**0.007**
Pre-Dawn (G)	1.85 ±0.66	1.10 ±1.26	**-0.52**	**0.003**	-0.05 ±0.51	-0.57 ±1.02	**-0.39**	**0.033**
Pre-Dawn (B)	1.27 ±0.65	0.53 ±1.19	**-0.51**	**0.004**	-0.63 ±0.51	-1.14 ±0.96	**-0.38**	**0.040**
Dawn (R)	1.67 ±0.34	1.22 ±0.95	-0.25	0.152	0.21 ±0.34	-0.29 ±0.96	-0.27	0.122
Dawn (G)	1.64 ±0.41	1.15 ±1.29	-0.18	0.328	0.18 ±0.41	-0.35 ±1.30	-0.19	0.288
Dawn (B)	1.09 ±0.42	0.64 ±1.20	-0.17	0.332	-0.37 ±0.43	-0.87 ±1.21	-0.19	0.291
Day (R)	4.69 ±0.28	5.21 ±0.26	**0.67**	**<0.001**	1.86 ±0.26	2.28 ±0.25	**0.57**	**<0.001**
Day (G)	5.01 ±0.30	5.52 ±0.27	**0.69**	**<0.001**	2.17 ±0.28	2.59 ±0.26	**0.60**	**<0.001**
Day (B)	4.63 ±0.29	5.08 ±0.27	**0.68**	**<0.001**	1.79 ±0.28	2.15 ±0.26	**0.58**	**<0.001**
Dusk (R)	1.64 ±0.44	1.78 ±0.37	-0.03	0.851	0.18 ±0.44	0.28 ±0.38	-0.08	0.667
Dusk (G)	1.66 ±0.41	1.93 ±0.39	0.20	0.276	0.20 ±0.41	0.43 ±0.39	0.15	0.408
Dusk (B)	1.14 ±0.47	1.51 ±0.47	0.26	0.151	-0.32 ±0.47	0.00 ±0.47	0.22	0.225
Post-Dusk (R)	2.46 ±0.52	1.88 ±0.76	**-0.60**	**<0.001**	0.30 ±0.40	0.09 ±0.54	**-0.37**	**0.033**
Post-Dusk (G)	2.39 ±0.48	1.87 ±0.72	**-0.61**	**<0.001**	0.24 ±0.37	0.09 ±0.48	**-0.35**	**0.046**
Post-Dusk (B)	1.77 ±0.50	1.26 ±0.77	**-0.5**	**<0.001**	-0.38 ±0.41	-0.52 ±0.54	-0.31	0.080
2h Post- Wake (R)	2.39 ±0.47	2.77 ±0.65	**0.38**	**0.029**				
2h Post-Wake (G)	2.37 ±0.55	2.88 ±0.75	**0.42**	**0.015**				
2h Post-Wake (B)	1.84 ±0.61	2.38 ±0.83	**0.39**	**0.024**				
2h Pre- Sleep (R)	2.49 ±0.43	2.69 ±0.68	0.07	0.694				
2h Pre-Sleep (G)	2.45 ±0.46	2.83 ±0.76	0.18	0.305				
2h Pre-Sleep (B)	1.86 ±0.54	2.36 ±0.84	0.24	0.175				

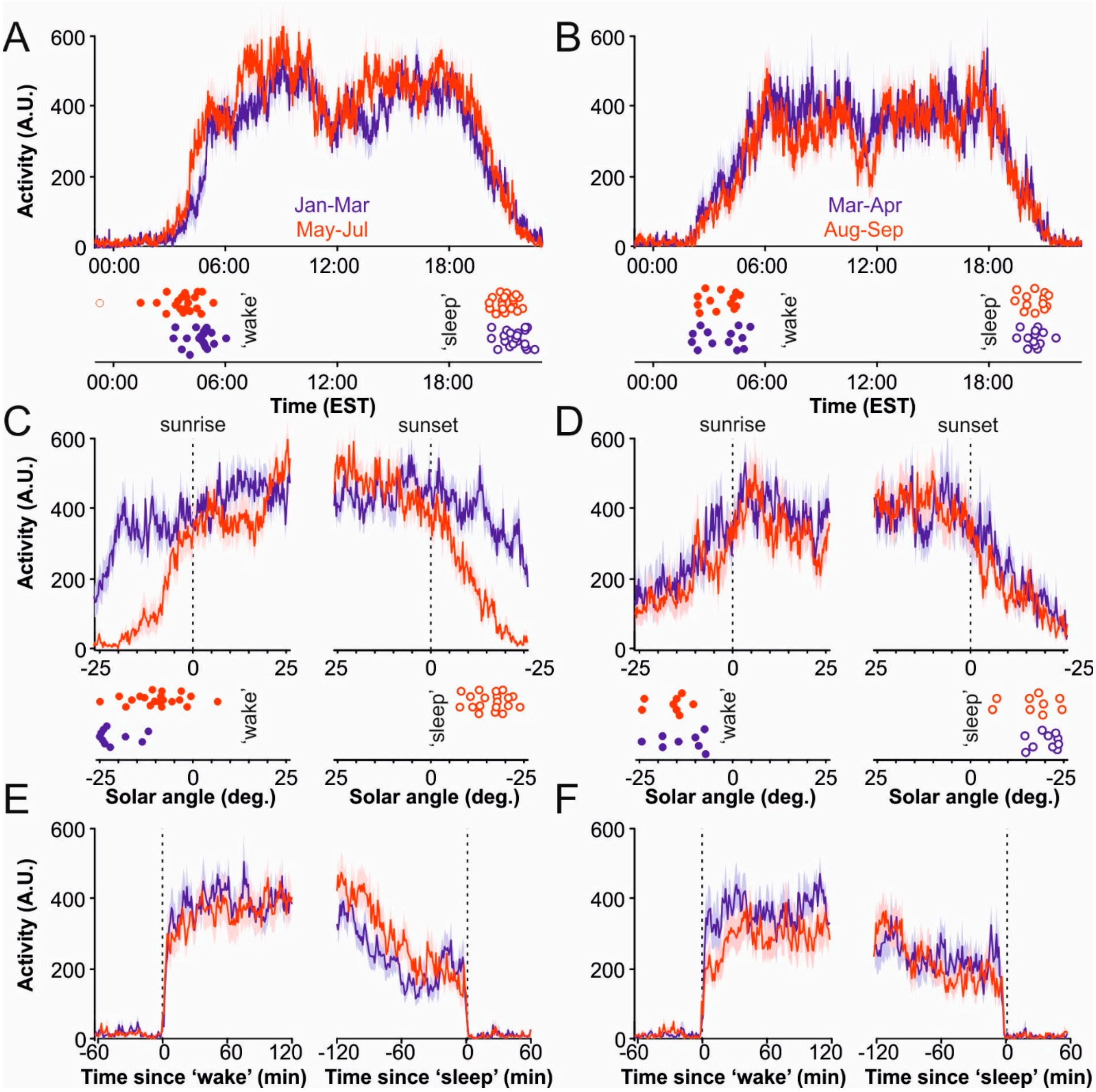

Figure S1. Activity timing in Old Order Amish subjects compared between epochs with large and smaller differences in photoperiod. (**A,B**) Mean ± SEM daily activity patterns for OOA subjects tested between Jan-Mar and May-Jul (A; n = 20, Δphotoperiod = 9–85 min) or Mar–Apr and Aug–Sep (B; n = 13, Δphotoperiod = 190–302 min). Lower panels indicates wake and sleep times for each subject. (**C,D**) Mean ± SEM activity patterns (upper panels) and wake/sleep times (lower panels) for subjects above as a function of sun position around dawn or dusk. (**E,F**) Mean ± SEM activity patterns for subjects above relative to actimetry defined wake and sleep timing.

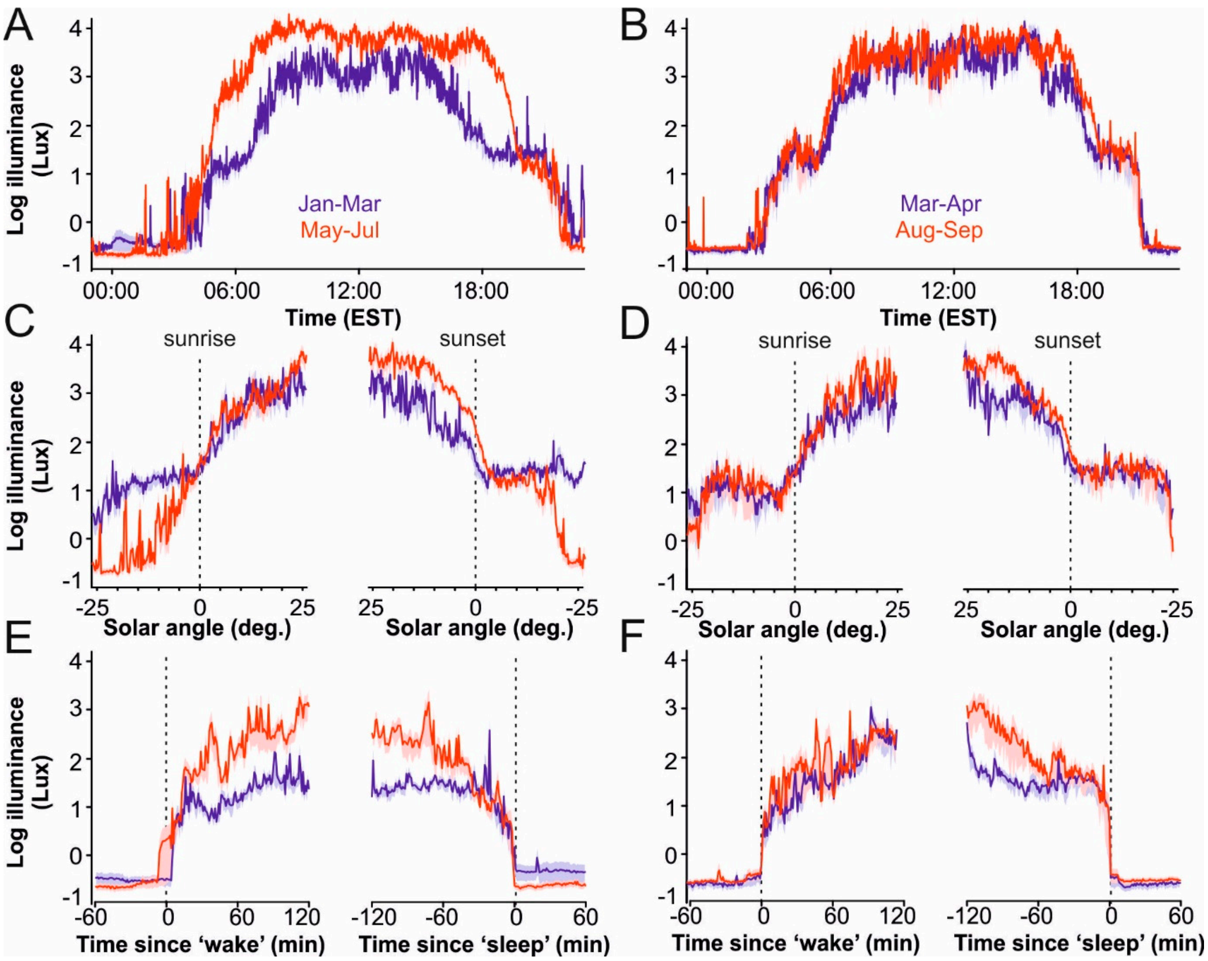

Figure 2. Photoperiod related differences in photopic light exposure in Old Order Amish subjects compared between epochs with large and smaller differences in photoperiod. (**A**,**B**) Mean ± SEM daily pattern of photopic light exposure for OOA subjects tested between Jan–Mar and May–Jul (A; n = 20, Δphotoperiod = 9–85 min) or Mar-Apr and Aug-Sep (B; n = 13, Δphotoperiod = 190–302 min). (**C**,**D**) Mean ± SEM pattern of photopic light exposure for subjects above as a function of sun position around dawn or dusk. (**E**,**F**) Mean ± SEM pattern of photopic light exposure for subjects above relative to actimetry defined wake and sleep timing.
